# An Externally Validated Nomogram for Predicting Lymph Node Metastasis of Presumed Stage I and II Endometrial Cancer

**DOI:** 10.3389/fonc.2019.01218

**Published:** 2019-11-14

**Authors:** Yangyang Dong, Yuan Cheng, Wenjuan Tian, Hua Zhang, Zhiqi Wang, Xiaoping Li, Boer Shan, Yulan Ren, Lihui Wei, Huaying Wang, Jianliu Wang

**Affiliations:** ^1^Department of Obstetrics and Gynecology, Peking University People's Hospital, Beijing, China; ^2^Department of Gynecologic Oncology, Fudan University Shanghai Cancer Center, Shanghai, China; ^3^Research Center of Clinical Epidemiology, Peking University Third Hospital, Beijing, China

**Keywords:** lymph node metastasis, endometrial cancer, nomogram, validation, net benefit

## Abstract

**Background:** Optimal management for endometrial cancer in patients with clinically negative lymph nodes is still under debate. Several prediction models for lymphatic dissemination of early-stage endometrial cancer have been developed. However, external validation is rare, and decision curve analysis has hardly been applied for these models.

**Objective:** To develop and validate a nomogram to predict lymph node metastasis of presumed stage I and II endometrial cancer.

**Study Design:** The prediction nomogram was developed by using multivariable logistic regression with data for 700 EC patients who underwent initial surgery from 2006 to 2017 at Peking University People's Hospital (training dataset), Beijing. External validation was performed in 727 eligible patients from Fudan University Shanghai Cancer Center (validation dataset), Shanghai.

**Results:** For the 700 women in the training dataset, the lymph node metastasis rate was 8.0% (56/700). Lymphovascular space invasion, histological grade, cervical stromal invasion, and myometrial invasion were independent prognostic factors in the training dataset. We generated a nomogram based on these pathological factors. To determine the clinical usefulness of our nomogram, we compared it with the Mayo criteria. For our nomogram, the area under the receiver operating characteristic curve (AUC) was 0.85 as compared with 0.63 for the Mayo criteria. In the validation dataset, the AUC was 0.78 as compared with 0.57 for the Mayo criteria. The nomogram was well-calibrated in both the training and validation datasets. At a 10% probability threshold, our nomogram decreased almost 29 unnecessary lymphadenectomies per 100 patients than the Mayo criteria without missing more metastatic disease.

**Conclusion:** We developed a nomogram to predict lymph node metastasis in patients with early-stage endometrial cancer in China. This prediction model may help clinicians in decision-making for patients with early-stage endometrial cancer, especially for the patient with incomplete surgery, reducing overtreatment, and medical costs.

## Introduction

Lymphadenectomy is controversial in early-stage endometrial cancer (EC). The net benefit for lymphatic dissemination in patients with early-stage endometrial cancer is difficult to estimate accurately. Furthermore, the contribution of lymphadenectomy to long-term survival of women with stage I and II endometrial cancer is ambiguous. Several studies have shown that lymphadenectomy was conducive to define the extent of disease lesions, guide adjuvant treatment, and improve prognosis (1–3). However, two large-scale randomized controlled trials hinted at opposite results. First, traditional lymphadenectomy could not improve disease-free survival or overall survival in EC patients (4, 5); second, the incidence of complications, such as intestinal obstruction, lymphocyst, deep venous thrombosis, and other surgical complications, increases with expansion of the scope of surgery (6–8). Moreover, the risk of lymph node metastasis in early-stage endometrial cancer is only about 10% (9). Whether lymphadenectomy leads to overtreatment of most patients with early endometrial cancer and increases additional medical costs remains a question.

Currently, most clinicians in China follow the criteria for judging low-risk groups without lymphadenectomy proposed by the Mayo clinic in 2000. The Mayo criteria classify endometrial cancer patients at low risk with the variables: endometrioid endometrial cancer, tumor diameter <2 cm, grade 1 or 2, and myometrial invasion (MI) <50%. Vargas et al. analyzed 19,329 patients with endometrioid adenocarcinoma in the SEER database whose lesions were confined to the uterus, retrospectively (10). According to Mayo criteria, 78.9% of the patients were at high-risk for nodal metastasis, and the lymph node metastasis rate was 6.4%. Thus, almost 70% patients without lymph node metastasis were over-treated. Therefore, accurately predicting lymph node metastasis is crucial for selecting an appropriate surgical approach especially for early-stage endometrial cancer.

Recently, some practitioners have developed some prediction models for lymphatic dissemination by using nomogram, a precise calculation system based on a statistical algorithm (11–15). However, external validation was rare. The net benefit of decision curve analysis in lymph node dissection for early-stage endometrial cancer has not been calculated in the above studies. In this study, we developed and externally validated a parametric model of pathological characteristics based on a mathematical algorithm for predicting lymph node metastasis in early-stage endometrial cancer. Moreover, to determine the clinical usefulness of the nomogram, we used decision curve analysis and compared it with the Mayo criteria.

## Methods

### Study Population

**Figure 1:**
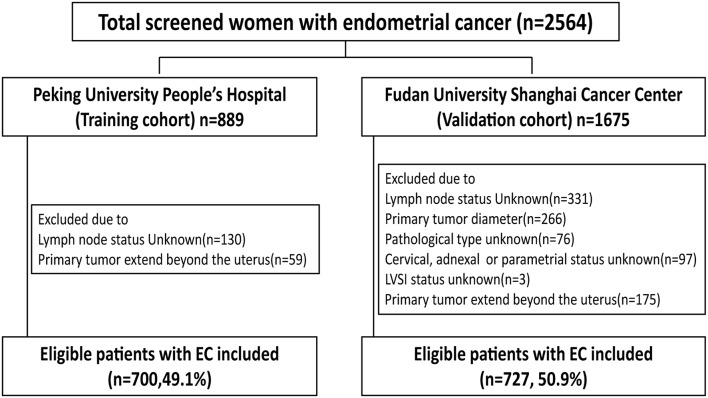
Flow chart of study participants in training and validation groups.

From January 2006 to December 2017, the data of all patients who had received primary surgical treatment for endometrial cancer were abstracted from the database of Peking University People's Hospital. The medical records were reviewed to determine age, menopausal status, surgical procedure, tumor diameter, depth of myometrial invasion (MI), cervical stromal invasion status, histological type, histological grade, lymphovascular space invasion (LVSI) status, and final lymph node status. Tumor diameter was defined as the largest of the three dimensions of the tumor measured on fresh tissue. A tumor was considered LVSI positive when tumor emboli were found within a space clearly lined by endothelial cells. Endometrioid and mucinous carcinomas were graded using the FIGO system. Typical type II endometrial cancer (serous and clear cell carcinomas) were not graded in our hospital, and all serious and clear cell carcinomas were considered grade 3. Inclusion criteria were endometrial cancer with final 2009 FIGO stages I, II, and IIIC cancer (with primary tumor confined to the uterus). Exclusion criteria were: patients who received neoadjuvant chemotherapy, patients with other primary synchronous malignancies, and patients with incomplete medical records. The group of 700 eligible women with early-stage endometrial cancer in Peking University People's Hospital between 2006 and 2017 was used to develop a nomogram. The group of 727 eligible patients with endometrial cancer from Fudan University Shanghai Cancer Center between 2006 and 2016 was used to validate the nomogram. The excluded patients in each group were detailed in Figure 1. The present study was approved by the ethics committee of Peking University People's Hospital and Fudan University Shanghai Cancer Center.

### Statistical Analysis

We estimated the sample size based on the area under the receiver operating characteristic curve (AUC). The AUC of our nomogram was estimated to be higher than 0.7. According to published articles, the risk of lymph node metastasis in early-stage endometrial cancer was about 10%, so we set the ratio between negative and positive cases as 9, with alpha error as 5% and test power as 95%. Therefore, our study should include at least 29 participants with lymph node metastasis and 261 participants without lymph node metastasis, which yielded a total sample size of 290 participants. Comparison of the training and validation cohorts involved chi-square or Fisher exact test for categorical variables.

The nomogram was developed using univariate and multivariate logistic regression model. All parameters that were significantly associated with lymph node metastasis in univariate analysis were entered in the full model. Candidate variables with *P* values <0.05 were selected using a backward stepwise selection from the full multivariate model. The selected variables were used to develop the nomogram.

The nomogram performance was assessed in both the training and validation groups by calculating discrimination and calibration. Discrimination is the ability to differentiate between women with lymph node metastasis and women without lymph node metastasis, and it is measured by AUC. Calibration is the agreement between the observed outcome frequencies and the predicted probabilities, and it is estimated by the Hosmer-Lemeshow goodness-of-fit test. The AUC ranges from 0 to 1, and a model is considered to have a poor, fair, or good performance if the AUC lies between 0.5 and 0.6, 0.6, and 0.7 or is >0.7, respectively. Calibration is reflected with average errors. The optimal cutoff point of the nomogram based on the training set was estimated by Youden's J index (16).

Decision curve analysis was used to quantify the clinical usefulness of the model. The clinical net benefit of the model: one sums the benefits (true positives) and subtracts the harms (false positives) at each threshold probability (Pt) of the outcome, and was calculated as:

$$Net\;Benefit\;=\;\frac{\begin{array}{c} \;\\ Ture\;Positive\;Count \end{array}\;}{n}-\frac{\begin{array}{c} \;\\ \;False\;Positive\;Count \end{array}}{n}(\frac{Pt}{1-Pt})$$

The net reduction presents the reduction of patients in unnecessary intervention per 100 patients, and was calculated as:

$$Net\;Reduction=\frac{\begin{array}{c} \;\\ (Net\;Benefit\;of\;the\;model-Net\;Benefit\;of\;treat\;all)*Pt \end{array}\;}{\;(1-Pt)}*100$$

Decision curve analysis of the nomograms and Mayo criteria involved using the Decision Curve package of R software.

All analyses were performed in SPSS v22.0 and R software version 3.4.4 (https://www.r-project.org/), using the rms, presence/absence, decision curve packages. *P* < 0.05 was considered statistically significant.

## Results

### Patients and Variables

The training cohort consisted of 700 eligible women with early-stage EC who underwent lymph node dissection (Figure 1). The external validation cohort included 727 patients. For the 700 women in the training cohort, the lymph node metastasis rate was 8.0% (56/700) vs. 11.8% (86/727) in the validation cohort (Table 1). The cohorts were similar in age, menopausal status, histological type, and LVSI. However, more women in the validation than training cohort had tumor diameter >2, grade 3, deep myometrial invasion, and cervical stromal invasion.

**Table 1:** Patient characteristics for the training and validation cohorts.

**Variables**	**Training cohort**	**Validation cohort**	***P-value***
	***N* %**	***N* %**	
	**700 49.1**	**727 50.9**	
**Age (year)**			0.056
<60	462 (66.0)	514 (70.7)	
≥60	238 (34.0)	213 (29.3)	
**Menopausal status**			0.439
Premenopause	231 (33.0)	226 (31.1)	
Postmenopause	469 (67.0)	501 (68.9)	
**Depth of myometrial invasion**			**<0.001**
<50%	544 (77.7)	494 (68.0)	
≥50%	156 (22.3)	233 (32.0)	
**Cervical stromal invasion**			**<0.001**
No	631(90.1)	574 (79.0)	
Yes	69 (9.9)	153 (21.0)	
**Histological grade**			**<0.001**
G1	250(35.7)	178 (24.5)	
G2	304(43.4)	371 (51.0)	
G3	146(20.9)	178 (24.5)	
**Histological type**			0.068
Endometrioid	637 (91.0)	640 (88.0)	
Non-endometrioid	63 (9.0)	87 (12.0)	
**Tumor diameter**			**<0.001**
<2	266(38.0)	177 (24.3)	
≥2	434(62.0)	550 (75.7)	
**Lymphovascular space invasion**			0.807
No	584 (83.4)	610 (83.9)	
Yes	116 (16.6)	117 (16.1)	
**Lymph node metastasis**			**0.016**
No	644 (92.0)	641 (88.2)	
Yes	56 (8.0)	86 (11.8)	

*Data are n (%) or mean± SD. Bold represents P-value of the variable is less than 0.05*.

### Factors Associated With Lymph Node Metastasis

On univariate logistic regression analysis, deep myometrial invasion, cervical stromal invasion, histological type, histological grade, tumor diameter, LVSI were associated with lymph node metastasis in the training cohort. All of these variables were analyzed by multivariable logistic regression analysis using backward method. Independent risk factors associated with lymph node metastasis in the training cohort were LVSI, grade, cervical stromal invasion, and myometrial invasion (Table 2). LVSI was a major predictor, with adjusted odds ratio (OR 6.5; 95% CI, 3.4–12.4; *P* < 0.001). Other predictors were depth of myometrial invasion ≥50% (OR 2.0; 95% CI, 1.1–3.9; *P* = 0.03), cervical stromal invasion (OR 3.5; 95% CI, 1.7–7.1; *P* < 0.001), grade 2 cancer (OR 2.9; 95% CI, 1.0–8.0; *P* = 0.046), and grade 3 cancer (OR, 3.6; 95% CI, 1.2–10.4; *P* = 0.021). The results of univariate and multivariable analysis in the validation cohort were in Table 3. LVSI, cervical stromal invasion and MI were also risk factors for lymph node metastasis in the validation cohort. But the effects of histological type and histological grade on lymph node metastasis slightly differed in the two cohorts.

**Table 2:** Univariate and multivariate analysis of predictors of metastatic lymph nodes in the training cohort.

**Variables**	**Univariate analysis**	***P–value***	**Multivariate analysis**	***P–value***
	**OR (95% CI)**		**Adjusted OR (95% CI)**	
**Age (year)**				
<60	1.0			
≥60	1.3 (0.7–2.2)	0.385		
**Menopausal status**				
Premenopause	1.0			
Postmenopause	1.3(0.7–2.3)	0.463		
**Depth of myometrial invasion**				
<50%	1.0		1.0	
≥50%	5.2(2.9–9.0)	**<0.001**	2.0 (1.1–3.9)	**0.03**
**Cervical stromal invasion**				
No	1.0		1.0	
Yes	7.4(4.0–13.8)	**<0.001**	3.5 (1.7–7.1)	**<0.001**
**Histological grade**				
G1	1.0		1.0	
G2	4.4 (1.7–11.6)	**0.003**	2.9 (1.0–8.0)	**0.046**
G3	10.6(4.0–28.3)	**<0.001**	3.6 (1.2–10.4)	**0.021**
**Histological type**				
Endometrioid	1.0		–	
Non–endometrioid	4.0(2.1–7.9)	**<0.001**	–	**–**
**Tumor diameter**				
<2	1.0		–	
≥2	4.0(1.9–8.6)	**<0.001**	–	**–**
**Lymphovascular space invasion**				
No	1.0		1.0	
Yes	11.6 (6.4–20.9)	**<0.001**	6.5 (3.4–12.4)	**<0.001**

*CI, confidence interval; OR, odds ratio. Bold represents P-value of the variable is less than 0.05*.

**Table 3:** Univariate and multivariate analysis of predictors of metastatic lymph nodes in the validation cohort.

**Variables**	**Univariate analysis**	***P–value***	**Multivariate analysis**	***P–value***
	**OR (95% CI)**		**Adjusted OR (95% CI)**	
**Age(year)**				
<60	1.0			
≥60	1.2 (0.7–1.9)	0.480		
**Menopausal status**				
Premenopause	1.0			
Postmenopause	0.7 (0.4–1.0)	0.073		
**Depth of myometrial invasion**				
<50%	1.0		1.0	
≥50%	3.9 (2.4–6.2)	**<0.001**	2.3 (1.4–3.8)	**0.002**
**Cervical stromal invasion**				
No	1.0		1.0	
Yes	3.8 (2.4–6.1)	**<0.001**	2.8 (1.7–4.7)	**<0.001**
**Histology grade**				
G1	1.0		–	
G2	2.2 (1.0–4.7)	**0.038**	–	**–**
G3	5.1 (2.4–10.9)	**<0.001**	–	**–**
**Histology type**				
Endometrioid	1.0		–	
Non–endometrioid	2.8 (1.6–4.9)	**<0.001**	2.0 (1.0–3.7)	**0.038**
**Tumor diameter**				
<2	1.0		–	
≥2	2.1 (1.1–4.0)	**0.019**	–	**–**
**LVSI**
No	1.0		1.0	
Yes	6.8 (4.2–11.0)	**<0.001**	4.4 (2.6–7.4)	**<0.001**

*OR, odds ratio; 95% CI, 95% confidence interval. Bold represents P-value of the variable is less than 0.05*.

### Development and Validation of the Prediction Model

**Figure 2:**
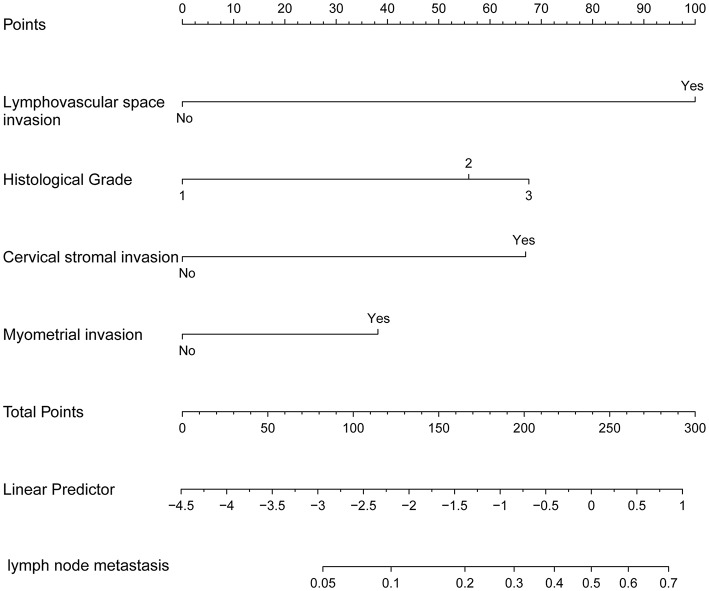
The nomogram of metastatic lymph node involvement calculated for each variable: lymphovascular space invasion (LVSI), grade, cervical stromal invasion, and myometrial invasion (MI).

**Figure 3:**
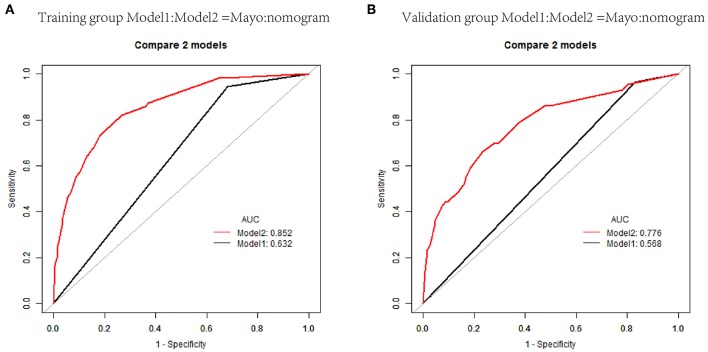
Receiver operating characteristic curves (AUCs) of nomogram and Mayo criteria for predicting lymph node metastasis in endometrial carcinoma in the training and validation cohorts. **(A)** AUCs in the training group. **(B)** AUCs in the validation group.

The nomogram constructed from the final multivariate model was presented in Figure 2. For a given patient, points were assigned to each of the predictor variables in the nomogram and a total score was derived from the sum of present variables. The total score corresponds to a predicted probability of lymph node metastasis. The performance of the final model was assessed through discrimination and calibration. The AUC for the nomogram was 0.85 (95% CI, 0.80–0.90) as compared with 0.63 (95% CI, 0.60–0.67) for the Mayo criteria in the training cohort (*P* < 0.001; Figure 3A). In the validation cohort, the AUC was 0.78 (95% CI, 0.72–0.83), as compared with 0.57 (95% CI, 0.54–0.59) for the Mayo criteria (*P* < 0.001; Figure 3B). The nomogram showed superior discrimination to the Mayo criteria both in the training and validation groups.

**Figure 4:**
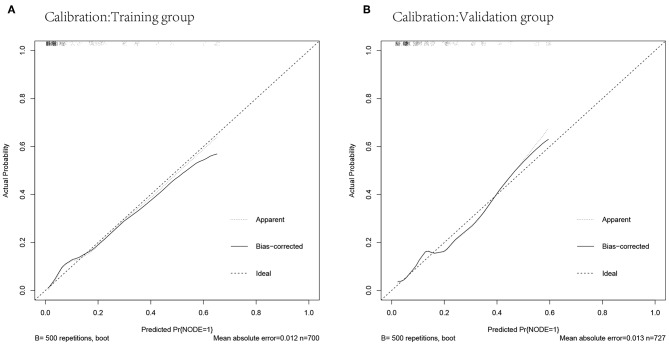
Calibrations of the nomogram for predicting lymph node metastasis in the training and validation cohorts. **(A)** Calibration in the training cohort. **(B)** Calibration in the validation cohort.

The predicted probabilities and the actual probabilities of lymph node metastasis in the training and validation groups are shown in the calibration plot (Figure 4). The nomogram was well-calibrated both in the training (mean absolute error = 0.012) and validation cohorts (mean absolute error = 0.013).

### Optimal Threshold of the Model

**Figure 5:**
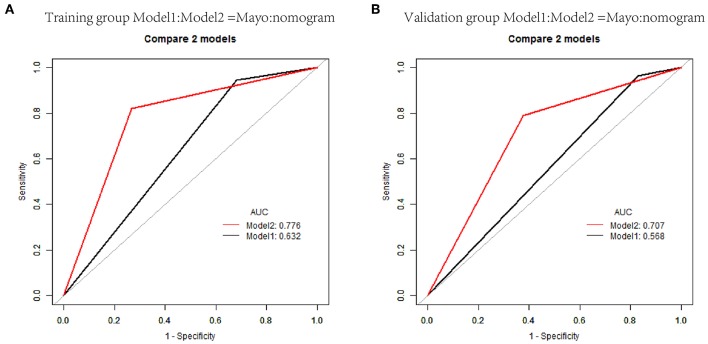
Receiver operating characteristic curves of classification based on nomogram score and Mayo criteria for predicting lymph node metastasis in endometrial carcinoma in the training and validation cohorts. **(A)** AUCs in the training group. **(B)** AUCs in the validation group.

Individualized scores for each patient were accurately calculated according the nomogram. Therefore, we used an optimal cut-off value that maximized the sum of sensitivity and specificity in the ROC curve. The best cut-off point was 80 points. According to the cut-off point, the patients, both in the training and validation cohorts, were divided into low-risk group (score <80 points) and high-risk group (score>80 points) according to the threshold. The performance comparison of nomogram stratification and Mayo criteria for predicting lymph node metastatic was verified. The nomogram showed a better discrimination than the Mayo criteria in both training (with AUC of 0.78, 95% CI, 0.72–0.83 vs. 0.63, 95% CI, 0.60–0.67; *P* < 0.001) and validation cohorts (with AUC of 0.71, 95% CI, 0.66–0.75, vs. 0.57, 95% CI, 0.54–0.59; *P* < 0.001; Figure 5). In the training cohort, the lymph node metastasis rates were 2.1 and 21.0% in low risk-group and high-risk group according to the nomogram, and those were 1.4 and 10.8% according to the Mayo criteria. In the validation cohort, the lymph node metastasis rates were 4.3 and 22% in low-risk group and high-risk group according to the nomogram, and those were 2.7 and 13.5% according to the Mayo criteria (Tables 4, 5).

**Table 4:** Performance of the Mayo criteria and nomogram scoring system in predicting metastatic lymph nodes in the training cohort.

**Performance parameter**	**Model**		**Comparison**
	**Mayo**	**Nomogram**	**Nomogram vs. Mayo**
AUC	0.63	0.78	0.15^a^
Accuracy	0.37	0.74	0.37
Specificity	0.32	0.73	0.41
Sensitivity	0.95	0.82	−0.13
Positive-LR	1.39	3.06	1.67
Negative-LR	0.17	0.24	0.07
Positive-PV	0.11	0.21	0.10
Negative-PV	0.99	0.98	−0.01

AUC, area under the receiver operating characteristic curve; LR, likelihood ratio; PV, predictive value;

a *P < 0.001 for comparing AUCs*.

**Table 5:** Performance of the Mayo and nomogram scoring system in predicting metastatic lymph nodes in the validation cohort.

**Performance parameter**	**Model**		**Comparison**
	**Mayo**	**Nomogram**	**Nomogram vs. Mayo**
AUC	0.56	0.71	0.15^a^
Accuracy	0.26	0.64	0.38
Specificity	0.17	0.62	0.45
Sensitivity	0.96	0.79	−0.17
Positive-LR	1.62	2.09	0.47
Negative-LR	0.21	0.34	0.13
Positive-PV	0.14	0.22	0.08
Negative-PV	0.97	0.96	−0.01

AUC, area under the receiver operating characteristic curve; LR, likelihood ratio; PV, predictive value;

a *P < 0.001 for comparing AUCs*.

### Decision Curve Analysis and Net Benefit

**Figure 6:**
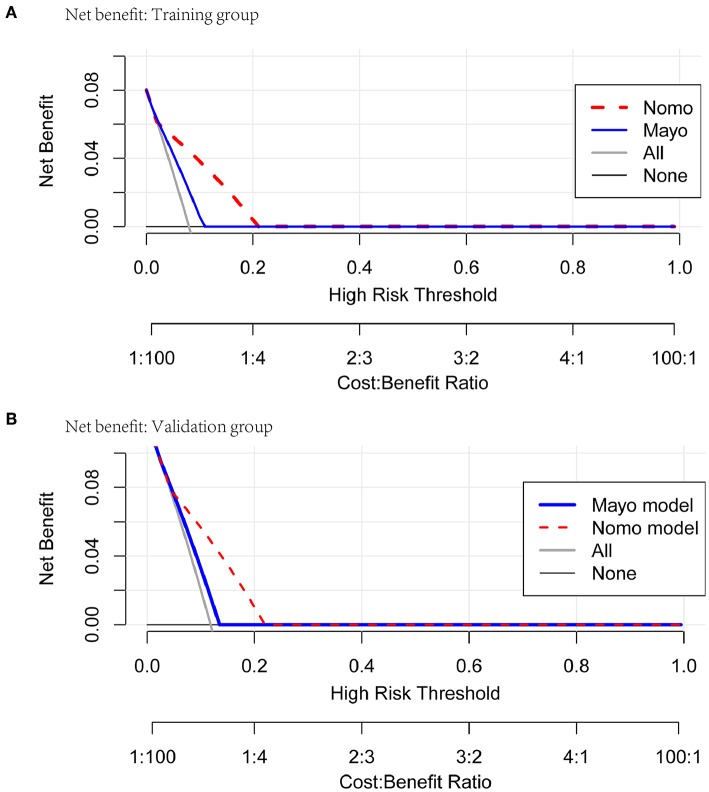
Net benefit curves for classification based on nomogram score as compared with the Mayo criteria in the training and validation cohorts.

Comprehensive comparative analysis, combined with a net benefit curve, showed that with threshold probability 3%~20%, the nomogram increased the net benefit by 0.2~3.5 per 100 patients in the training cohort (Table 6, Figure 6A). We observed similar results in the validation cohort (Table 7, Figure 6B). At a 10% probability threshold, our nomogram led to 3.8 positive result per 100 patients without an increase in the number of false-positive results, while Mayo can only lead to 0.6 positive patient. At this probability threshold, the nomogram also decreased 28.8 unnecessary interventions per 100 patients without missing more metastatic disease than the Mayo criteria.

**Table 6:** Net benefit of the Mayo criteria and nomogram scoring system in predicting metastatic lymph nodes in the training cohort.

**Threshold (%)**	**Net benefit**		**Advantage of nomogram**	
	**Mayo(%)**	**Nomogram(%)**	**Net benefit (%)**	**Net reduction (%)**
0	8.0	8.0	0.0	0.0
1	7.1	7.1	0.0	0.0
2	6.3	6.1	−0.2	−9.8
3	5.6	5.8	0.2	6.5
4	5.0	5.5	0.5	12.0
5	4.3	5.3	1.0	19.0
6	3.6	5.0	1.4	21.9
7	2.8	4.7	1.9	25.2
8	2.1	4.4	2.3	26.5
9	1.4	4.1	2.7	27.3
10	0.6	3.8	3.2	28.8
11	0.0	3.5	3.5	28.3
12	0.0	3.2	3.2	23.5
13	0.0	2.9	2.9	19.4
14	0.0	2.5	2.5	15.4
15	0.0	2.2	2.2	12.5
16	0.0	1.9	1.9	10.0
17	0.0	1.5	1.5	7.3
18	0.0	1.1	1.1	5.0
19	0.0	0.8	0.8	3.4
20	0.0	0.4	0.4	1.6

**Table 7:** Net benefit of the Mayo criteria and nomogram scoring system in predicting metastatic lymph nodes in the validation cohort.

**Threshold (%)**	**Net benefit**		**Advantage of nomogram**	
	**Mayo (%)**	**Nomogram (%)**	**Net benefit (%)**	**Net reduction (%)**
0	11.8	11.8	0.0	0.0
1	10.9	10.9	0.0	0.0
2	10.0	10.0	0.0	0.0
3	9.0	9.1	0.1	3.2
4	8.4	8.2	−0.2	−4.8
5	7.6	7.6	0.0	0.0
6	6.7	7.2	0.5	7.8
7	5.9	6.8	0.9	12.0
8	5.1	6.5	1.4	16.1
9	4.2	6.1	1.9	19.2
10	3.3	5.7	2.4	21.6
11	2.4	5.2	2.8	22.7
12	1.4	4.8	3.4	24.9
13	0.5	4.4	3.9	26.1
14	0.0	3.9	3.9	24.0
15	0.0	3.5	3.5	19.8
16	0.0	3.0	3.0	15.8
17	0.0	2.5	2.5	12.2
18	0.0	2.0	2.0	9.1
19	0.0	1.5	1.5	6.4
20	0.0	1.0	1.0	4.0

## Discussion

Although lymph node status is a very important prognostic factor, the incidence of lymph node metastasis is only about 10% in clinically suspected early-stage endometrial cancer (9), and the published results of two randomized trials did not show survival benefit for routine lymphadenectomy in early-stage endometrial cancer (4, 5). In addition, surgical morbidities increase by performing lymphadenectomy. Hence, systematic lymphadenectomy has become a matter of debate in early-stage endometrial cancer. Several risk classifications are used to identify subsets of patients with lower risk of lymph node metastasis. The Mayo criteria was mostly used in China. However, according to the Mayo criteria, the lymph node metastasis rate for the high-risk group was 6.4%, more than 70% patients without lymph node metastasis were over-treated (10). The key to solve this dilemma is how to accurately identify potential lymph node metastasis. Sentinel lymph-node biopsy with less invasive intervention is being adopted in surgical treatment of EC (17). However, the significance of such biopsy in assessing lymph node metastasis has been found to be limited (18, 19), and the prognostic value of the intervention is still under investigation. Based on this background information, we aimed to construct a prediction model that could identify patients who required systematic lymphadenectomy.

Clinicopathological variables, such as histological type and grade, tumor diameter, depth of myometrial invasion, cervical stromal invasion, LVSI, CA-125 have been reported to be associated with lymph node metastasis. However, none of these parameters can accurately identify patients with lymph node metastasis individually. Several authors developed models to predict lymph node metastasis by combining multiple indicators (11–15) (Table 8).

**Table 8:** Summary of published nomograms for predicting lymph node metastasis in endometrial cancer.

		**Taşkin** **(11)**	**Pollom (12)**	**Bendifallah (13)**	**Kang** **(14)**	**Alhilli** **(15)**
Participant		Stage 1–4	Stage 1–4	Stage 1–2	Stage 1–4	Stage 1–3
Sample size		279	296	523	397	883
Participant	LVSI	✓	✓	✓	✓	✓
	Grade			✓	✓	✓
	MI		✓	✓	✓	✓
	Cervical	✓	✓			✓
	Histology type	✓				
	CA-125	✓			✓	
	Diameter		✓	✓		✓
C-index		0.92	0.83	0.83	0.87	0.88
Validation		Absent	Absent	Absent	Absent	Present

Aihilli et al. established a model for predicting lymph node metastasis in 883 women with early-stage endometrioid EC that included MI, grade, primary tumor diameter, cervical stromal invasion and LVSI (15). The internal validation of the nomogram showed a good discrimination (AUC = 0.88), but the author did not validate the model to assess the applicability and to an independent population. Bendifallah et al. performed an external validation of the prediction model in 322 patients with early-stage endometrioid EC (20). They found the model had a fair discrimination with a C-index of 0.65. Then, Bendifallah et al. developed a nomogram with data for 523 early-stage EC women based on four factors including grade, LVSI, MI, and tumor diameter, with a C-index of 0.83 (13), but there was no external validation either.

There are three other nomograms to predict lymph node metastasis in endometrial cancer. Kang et al. analyzed the clinical data for 307 cases of stage I-IV endometrial cancer with 31 cases (11.1%) had lymph node metastasis. On multivariate regression analysis, LVSI, grade, MI and tumor diameter were selected as high-risk factors of lymph node metastasis. The C-index of the model was 0.87, which had very good discrimination (14), but the number of cases was limited and external validation was not performed. Pollom et al. developed a prediction model using LVSI, MI, cervical stromal invasion, and tumor diameter in 296 patients with stage I-IV endometrial cancer, the scoring system had good validation in an internal cohort, with AUC of 0.83 (12). Taşkin et al. analyzed the clinical data for 279 cases of stage I-IV endometrial cancer. On multivariate regression analysis, CA125, histological type, cervical interstitial involvement and LVSI were selected as high-risk factors of lymph node metastasis. The C-index of the model was 0.92, which had a very good discrimination (11), but the number of cases was limited and external validation was not performed either. Owing to small sample size, these three nomograms included early and advanced endometrial cancer and no external validation was performed.

In our study, LVSI, MI, grade, and cervical stromal invasion were selected to construct the nomogram using multivariable logistic regression analysis. These four high-risk factors predicting lymph node metastasis were also present in previous studies. Seven hundred and twenty-seven patients with endometrial cancer, from Fudan University Shanghai Cancer Center between 2006 and 2016, were used to validate the nomogram. Our data showed that the discriminations were good both in the training cohort (AUC = 0.85) and in the validation cohort (AUC = 0.78). As the Mayo criteria was mostly used in China, we compare our nomogram with it. Our nomogram was better than the Mayo criteria. The clinical decision-making curve shows that, within a threshold probability ≥3.0%, patients will receive more net benefit from application of the nomogram than Mayo criteria. We use 80 points as the cut-off point based on Youden's J index. The patients were divided into low-risk group (score <80 points) and high-risk group (score>80 points). The performance comparison of nomogram stratification and Mayo criteria for predicting lymph node metastatic was verified. The nomogram showed a better discrimination than the Mayo criteria in both training (with AUC of 0.78, 95% CI, 0.72–0.83 vs. 0.63, 95% CI, 0.60–0.67; *P* < 0.001) and validation cohorts (with AUC of 0.71, 95% CI, 0.66–0.75, vs. 0.57, 95% CI, 0.54–0.59; *P* < 0.001). Thirty-two percent of the patients were classified as high-risk group according to the nomogram stratification, compared with 70 percent based on the Mayo criteria. The lymph node metastasis rates were 2.1 and 21.0% in low risk-group and high-risk group, according to the nomogram, while those were 1.4 and 10.8% according to the Mayo criteria. At a 10% probability threshold, our nomogram led to 3.8 positive result per 100 patients without an increase in the number of false-positive results, while Mayo only can lead to 0.6 positive patient. On the other hand, at this probability threshold, the nomogram can decrease 28.8 unnecessary interventions per 100 patients without missing more metastatic disease than the Mayo criteria.

There are some limitations in the current research. First, our model was established by applying postoperative indicators, so it is mainly used to evaluate whether lymphadenectomy should be performed after incomplete surgery. Second, our data were not subdivided because of the limited number of cases. Lymph node metastasis was not divided into pelvic and para-aortic lymph node metastasis in the cohorts. Type I and II were not considered separately. In addition, the research was retrospective study, and prospective external validation was needed to verify the promotion and application of the model.

## Conclusion

In conclusion, our research involved the development and external validation of a feasible prediction model of lymph node metastasis in early-stage endometrial cancer. The nomogram to predict lymph node metastasis had better accuracy and net benefit than the Mayo criteria. Calibrations were relatively good between the two institutions. The newly developed nomogram can preferably guide decision-making for surgery for women with early-stage EC to avoid overtreatment and unnecessary costs.

## Data Availability Statement

The datasets generated for this study are available on request to the corresponding author.

## Ethics Statement

Written informed consent was obtained from the individual(s) for the publication of any potentially identifiable images or data included in this article.

## Author Contributions

JW and HW mainly designed this project and revised the manuscript. YD and YC were responsible for collecting data from Peking University People's Hospital, sorting up and statistical data, writing and revising the manuscript. HZ mainly gave guidances in data statistics and R language programming. WT, BS, and YR mainly established the database of endometrial cancer form Fudan University Shanghai Cancer Center. ZW, XL, and LW gave the suggestions in clinical value and application of the predictive model.

### Conflict of Interest

The authors declare that the research was conducted in the absence of any commercial or financial relationships that could be construed as a potential conflict of interest.
